# Biofunctional Magnetic Carbon Nanohybrid for Fast Removal of Methyl Blue from Synthetic Laboratory Effluent

**DOI:** 10.3390/ma18133168

**Published:** 2025-07-03

**Authors:** Juan A. Ramos-Guivar, Melissa-Alisson Mejía-Barraza, Renzo Rueda-Vellasmin, Edson C. Passamani

**Affiliations:** 1 Grupo de Investigación de Nanotecnología Aplicada para Biorremediación Ambiental, Energía, Biomedicina y Agricultura (NANOTECH), Facultad de Ciencias Físicas, Universidad Nacional Mayor de San Marcos, Lima 15081, Peru; 2 Departamento de Física, Universidade Federal do Espírito Santo, Vitória 29075-910, Brazil

**Keywords:** aqueous dye remediation, magnetic separation, polyphenol-mediated synthesis, FTIR adsorption mechanism, surface functionalization, multiwall carbon nanotubes

## Abstract

The contamination of aquatic systems by industrial dyes, particularly methylene blue (MB), presents a significant environmental challenge due to their chemical stability and toxicity. In this study, the development and application of a novel magnetic nanohybrid comprising multiwall carbon nanotubes (MWCNTs) functionalized with maghemite (γ-Fe_2_O_3_) nanoparticles biosynthesized using *Eucalyptus globulus* extract (denoted MWNT-NPE) is reported. The material was thoroughly characterized by X-ray diffraction (XRD), Brunauer–Emmett–Teller (BET), Vibrating Sample Magnetometer (VSM), and Fourier-Transform Infrared (FTIR) techniques, revealing high crystallinity, mesoporosity, and superparamagnetic behavior. The MWNT-NPE exhibited exceptional MB adsorption performance under optimized conditions (pH 6, 0.8 g L^−1^ dose, 40 min equilibrium), achieving a maximum adsorption capacity of 92.9 mg g^−1^. Kinetic analysis indicated chemisorption and physisorption regimes depending on MB concentration, with the pseudo-second-order and Freundlich isotherm models providing the best fits of experimental data. FTIR spectroscopy demonstrated that the removal mechanism involves π–π stacking, hydrogen bonding, and electrostatic interactions between MB molecules and the composite’s surface functional groups. Notably, the magnetic nanohybrid retained over 98% removal efficiency across five regeneration cycles and successfully removed MB from synthetic effluents with efficiencies exceeding 91%. These findings highlight the synergistic adsorption and magnetic recovery capabilities of the bio-functionalized hybrid system, presenting a sustainable, reusable, and scalable solution for industrial dye remediation.

## 1. Introduction

Water, an essential resource for life on Earth, faces increasing threats due to the various forms of industrial pollution that have progressively increased since the industrial Revolution in the 18th Century. Among the most relevant pollutants are dyes, present in large quantities in wastewater from several industries such as textiles, leather, paper, plastics, dyeing, printing, cosmetics, etc. These types of pollutants are difficult to manage and adversely affect the aquatic environment that receives them [1,2]. Even at very low concentrations, e.g., less than 1 mg L^−1^ for some, dyes negatively impact water, affecting its quality, altering gas solubility, and reducing photosynthetic activity due to the decreased penetration of light into the deeper layers of lakes, rivers, and other bodies of water [3]. Due to their low costs, most dyes used in the industry are synthetic with aromatic rings, which, due to their high chemical stability, hinder and slow down their environmental degradation [4]; this observation exacerbates their impacts as they are not usually removed during conventional water treatment processes, persisting in temperature, light, water, and other substances, including soap and detergents [5].

Methylene Blue (MB) is a heterocyclic aromatic chemical compound with the chemical formula C_16_H_18_N_3_SCl, showing a maximum light absorption around 665 nm, and is one of the most studied dyes due to its positive and negative aspects [6,7]. Although MB presents therapeutic benefits in controlled clinical contexts, such as antioxidant, neuroprotective, and antimicrobial properties [7], its release into water bodies as industrial waste turns it into a toxic contaminant, capable of causing adverse effects like ocular burns, gastrointestinal tract irritation with symptoms of nausea, vomiting, and diarrhea if ingested, as well as methemoglobinemia, cyanosis, seizures, and dyspnea if inhaled [8]. Similarly, in photosynthetic organisms, concentrations as low as 0.1 mg L^−1^ of MB are sufficient to inhibit their growth, reduce pigment, and decrease protein content in the microalgae *Chlorella vulgaris* and *Spirulina platensis* [9]. In this context, the development of efficient methods for the removal of MB in water bodies not only represents an urgent environmental need, but also a priority for the preservation of human health, considering the toxic effects that this compound can generate.

Among all the treatments used for dye removal in water, such as photocatalytic degradation, sonochemical degradation, enhanced micellar ultrafiltration, cation exchange membranes, electrochemical degradation, adsorption/precipitation processes, and adsorption on activated carbon, adsorption was considered a simple and economical method for the removal of dyes from water and wastewater [1], mainly because it avoids the formation of secondary pollutants that can be generated during the oxidation or degradation processes of MB [10].

Also, recent advances have shown that functionalized carbon nanofibers (CNFs), especially those doped with heteroatoms such as nitrogen and oxygen, exhibit high adsorption capacity for organic pollutants in aqueous media. For instance, Mishakov et al. [11] demonstrated the synthesis of N- and O-doped CNFs via the decomposition of chlorinated hydrocarbons over self-organizing Ni-Pd catalysts. These CNFs achieved promising performance in removing 1,2-dichlorobenzene from water, attributed to their high surface area (up to 470 m^2^/g), enhanced porosity, and tailored surface chemistry. The incorporation of heteroatoms improves electronic properties and binding affinity to target contaminants, supporting their application in water purification technologies.

On the other hand, carbon nanotubes (CNTs) represent a highly attractive material for adsorption processes, thanks to their high surface-to-volume ratio, high thermal and chemical stability, easy functionalization through the attachment of magnetic nanoparticles (NPs) or chemical groups on their surface, and well-defined adsorption sites [12]. CNTs are classified into single-walled carbon nanotubes (SWCNTs) and multi-walled carbon nanotubes (MWCNTs). In particular, activated carbon is the most commonly used method for removing dyes by adsorption based on its highly porous structure and large surface area of between 500 and 2000 m^2^ g^−1^ [13,14], but, although the activated carbon is usually more economical than MWCNTs. However, the effective reuse of the MWCNTs, after they are exhausted significantly, improves their cost-effectiveness [15].

An issue that still remains an ecological barrier when the CNTs are applied in adsorption processes is their full recovery after their use in aqueous solutions, i.e., a complicated issue based on their small size and dispersion, posing a potential environmental risk. For this reason, in recent years, magnetic separation technology has been combined with the adsorption process, widely used for the removal of dyes in wastewater [16], to reduce this problem. An example is the study conducted by Parlayıcı and Pehlivan [17], who synthesized hydrogel-based beads composed of MWCNTs, TiO_2_, and chitosan and achieved an absorption capacity of 80.65 mg g^−1^ for MB. Similarly, Boukhalfa et al. [15] developed an alginate material functionalized with MWCNTs, achieving a maximum adsorption capacity of 905.5 mg g^−1^ for the same compound. In that sense, little has been researched on the use of green synthesis methods in the preparation of adsorbents, especially regarding their effect on the stability of MWCNTs functionalized with magnetic NPs. Moreover, this approach would not only reduce the environmental impact found in conventional synthesis, but it could also enhance the functional properties of the material, such as its adsorption capacity, size, and reusability, and, of course, increase the power of removing the material from the effluent after its treatment.

Underthese considerations, the present study proposes the use of *Eucalyptus globulus* (rich in polyphenols) in the synthesis and enhancement of physicochemical properties of MWCNTs functionalized with magnetic NPs to enhance MB removal in wastewaters. Thus, magnetic nanocarbon hybrids have been prepared, characterized by several techniques, and tested in the removal process of MB, showing a removal capacity of 92.9 mg g^−1^ under pH 6, a dose of 0.8 g L^−1^, and for an equilibrium time of 40 min. In addition, after several adsorption tests, the hybrid material still shows efficiency higher than 91%, demonstrating its potential for technological applications in magnetic remediation of contaminated water.

## 2. Materials and Methods

### 2.1. Materials

The biosynthesis process involved the use of FeSO_4_•7H_2_O (Merck KGaA, Darmstadt, Germany, purity > 99.5%) and FeCl_3_ (Sigma Aldrich, Burlington, MA, USA, 97% pure) in a molar ratio of 1:2 with the addition of NH_4_OH (Sigma Aldrich, Burlington, MA, USA, 28% *v*/*v*). The multiwall carbon nanotubes (MWCNTs) were obtained from Chengdu Zhongke Times Nano Energy Tech Co., Ltd., Chengdu, China, with the following technical data sheet: external diameter, 20–30 nm; internal diameter, 5–10 nm; length, 10–30 µm; purity > 98%; and specific surface area > 110 m^2^/g and synthesized by chemical vapor deposition. Methylene blue (MB) in powder was purchased from MERCK (Rahway, NJ, USA).

### 2.2. Extract Preparation and Adsorbent’s Biosynthesis

**Scheme 1:**
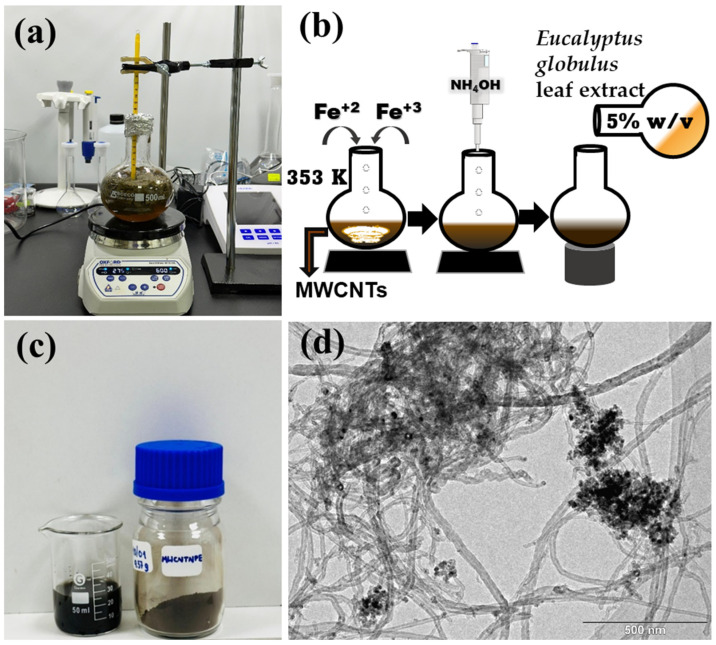
(**a**) Preparation of *Eucalyptus globulus* leaf extract at 5% *w*/*v*, (**b**) biosynthesis process, and (**c**) final biosynthesized nanohybrid at liquid (left) and powder (right) obtained and labeled as MWNT-NPE. (**d**) Representative TEM image of the MWNT-NPE. Bar scale of 100 nm for MWNT-NPE.

The *Eucalyptus globulus* leaf extract was prepared using the decoction method [18]. For that purpose, eucalyptus leaves were washed with ultrapure water, dried, and cut. The 5% *w*/*v* extract concentration was obtained by adding 20 g of eucalyptus leaves to 400 mL of ultrapure water. They were heated and boiled for 40 min. The resultant extract was further cooled to 300 K and filtered using Whatman #1 paper. The extract solution was designated E and stored at 278 K. Scheme 1a depicts the extract preparation, while Scheme 1b shows the biosynthesis process, which includes the addition of iron salts (5.2 g of FeSO_4_•7H_2_O and 6.0 g of FeCl_3_) into the MWCNTs dispersion at 353 K. Then, the addition of 15 mL of ammonium hydroxide was employed. The black dispersion was left to react for 30 min. After that, the dispersion was left to cool down to 300 K and washed several times till pH = 7. Thereafter, the 5% *w*/*v* E extract was added to the dispersion and heated at 353 K for 24 h. The process of washing was repeated till a neutral pH was reached. After that, the solid was obtained by drying the powders at 353 K; see Scheme 1c. Finally, a TEM representative image of the MWNT-NPE nanohybrid is shown in Scheme 1d.

### 2.3. Characterization

The measurements were performed using a Rigaku Ultima IV diffractometer (Tokyo, Japan) with CuK_α_ radiation (λ = 1.5418 Å), operating at 40 kV and 30 mA. The Bragg-Brentano geometry was used, scanning a 2*θ* range from 10° to 80° for the MWNT-NPE sample. An angular step of 0.01° and a counting time of 10 s per step were employed to ensure high resolution and detailed data acquisition of the X-ray diffractograms. Phase identification was carried out using the Match! v3 software, through which CIF files #9006316 and #1011060 were obtained from the Crystallographic Open Database (COD), corresponding to maghemite and the carbon nanotube phase, respectively, for the MWNT-NPE sample. De Keijser’s microstructural size model was used to find microstructural parameters [19] using the Instrumental Resolution File (IRF file) for the CO_2_ standard sample. The sample morphologies were obtained using a JEOL JEM-2000 FX instrument (Tokyo, Japan) running at 200 kV. Magnetic characterization was conducted using zero-field cooling (ZFC) and field cooling (FC) protocols. Magnetization versus magnetic field (*M*(*H*)) curves were acquired at 300 K and 4 K using the vibrating sample magnetometer (VSM) module of a Physical Property Measurement System (PPMS Evercool II, Quantum Design, San Diego, CA, USA). The FC protocol was applied under a cooling field of 1 kOe and a sweeping field of ±60 kOe. For the MWNT-NPE sample, *M*(*H*) loops were recorded at a maximum applied field of ±60 kOe. The hydrodynamic diameter and zeta potential of the MWNT-NPE sample (used in dye adsorption studies) were determined using a Brookhaven NanoBrook 90 Plus PALS instrument (New York, NY, USA) in conjunction with the Particle Solution software (BIC v.3.6.0.7122). Nitrogen adsorption–desorption isotherms at 77 K were measured using a NOVA 600 analyzer (Anton Paar Peru S.A.C., Lima, Peru) after degassing the samples at 573 K for 3 h. Textural properties, including specific surface area (SSA) and pore size distribution, were assessed using the multi-point Brunauer–Emmett–Teller (BET) method and the density functional theory (DFT) model [20].

Attenuated total reflectance Fourier transform infrared spectroscopy (ATR-FTIR) was used to characterize the powder samples vibrationally. The Lyza 7000 spectrometer, manufactured by Anton Paar Peru S.A.C. (Lima, Peru), operated between 4000 and 400 cm^−1^ with a spectral resolution of 2 cm^−1^ (number of scans = 36).

### 2.4. MB Adsorption Experiments

First, the batch adsorption protocol was developed by correctly measuring the absorbance of the aliquots. Using kinetic times between 5 and 60 min, the adsorbent was magnetically decanted for 5 min using a neodymium magnet. However, smaller particles remained even after increasing the magnetic decantation time to 20 min. This caused the UV-Vis spectra to show an additional absorbance band located at ~460 nm, confirming the presence of magnetic NPs and affecting the correct determination of the MB absorbance, as it will be discussed in Section 3.2. For example, for 30 min, the absorbance was measured to be 0.32 with a small removal efficiency of 22%. But, when applying a double filtration process, the absorbance value was reduced to 0.06, resulting in a removal efficiency of 88%. Therefore, the adsorption protocol was carried out by introducing the separation magnetic process plus a double filtration process.

In the adsorption experiments, the MWNT-NPE sample was used with MB dye. It should be noted that all the mentioned experiments were carried out at 300 K, in duplicate, and at a speed of 500 rpm. In the adsorption kinetics tests, 20 mg of the MWNT-NPE sample was applied in 25 mL of MB solution (adsorbent dose of 0.8 g L^−1^), with initial concentrations of 3 mg L^−1^ and 40 mg L^−1^ at a pH of 6. To determine the equilibrium kinetic time and study the influence of initial concentration, 11–12 points were used in the range of 5 to 120 min. Regarding the influence of pH on adsorption, the solution of 20 mg L^−1^ MB solution was adjusted to different pH levels (2, 4, 6, 8, 10, and 12), applying the MWNT-NPE sample in the following conditions: dose of 0.8 g L^−1^ for 40 min.

To determine the adsorption isotherm, a working pH value of 6 was chosen at 300 K. For the 5–90 mg L^−1^ MB dye, the experimental conditions were an equilibrium time of 40 min with a dose of 0.8 g L^−1^.

Adsorbent doses were also evaluated using 25 mg, 30 mg, 60 mg, 90 mg, and 120 mg of MWNT-NPE in 30 mL of 40 mg L^−1^ MB dye solution for 40 min at pH = 6.

Each batch adsorption experiment was conducted with two replicates, and the results were presented as mean ± SD (standard deviation).

The reuse performance of the MWNT-NPE sample was evaluated over five regeneration cycles, using the following procedure. The sample used in the first cycle was washed with 0.1 M NaOH for 30 min. It was then washed four times, returned to neutral pH, dried at 338 K, and used a second time in the dye solution. This process was repeated until the fifth use. It should be noted that these experiments were carried out in triplicate and according to the optimal parameters established for 20 mg L^−1^ MB at a pH of 6 and a dose of 2 g L^−1^ for 40 min and at 300 K.

To determine and read the concentration for each experiment, a calibration curve was constructed for MB dye. This process involved the preparation of various concentrations of dyes from 2 to 8 mg L^−1^, followed by the measurement of absorbance over a wavelength range of 600 to 700 nm, using the AVANTES UV-visible spectrophotometer (Apeldoorn, the Netherlands). From these data, the respective calibration curve for MB dye was derived. At the end of each experiment, a neodymium magnet was placed at the base of the vessel to separate the MWNT-NPE, characterized by its magnetic pulling force, to easily treat the aliquot. The resulting sample was transferred to a plastic cuvette and subjected to UV-visible spectrophotometer readings. Absorbance curves were recorded, enabling the concentration to be calculated. In that sense, the removal efficiency, %*R*, and the adsorption capacity $q_{t}$ were respectively calculated using Equations (1) and (2):

(1) $$\%R=\frac{C_{i}-C_{t}}{C_{i}}\times 100\%$$

(2) $$q_{t}(mg{\;g}^{-1})=\left(C_{i}-C_{t}\right)\times \frac{V}{m}$$

where $C_{i}$ is the initial dye concentration, $C_{t}$ is the dye concentration at a certain time, V (mL) is the volume of the solution, and m (mg) is the amount of adsorbent.

The obtained kinetic and isothermal data were fitted using various adsorption models reported in the literature. Indeed, these models, including the error analysis, are described in the Supplementary Materials; see Sections S1 and S2.

### 2.5. Preparation of the Synthetic MB Effluent

Approximately 0.1 g of MB dye was added to 1 L of water to obtain 100 mg L^−1^ of concentration. Then, 15 g of starch was added to the solution till the texture was not too thick or watery. After that, the cotton fiber was soaked in ultrapure water and dried until it was damp. Then, the damp fiber was placed in the 100 mg L^−1^ dye solution for 10 min. The remaining solution was labeled as Effluent-1 solution (impregnating solution, MB dye concentration of 2.2 mg L^−1^). After that, the fiber was located in an autoclave (BIOBASE, model BKM-P24, Jinan, China) and heated to 120 °C (~0.1 MPa) for 30 to 60 min. After this process, the fiber was removed and placed in 4 L of cold water containing 5% *w*/*v* acetic acid for 10 min. The fiber was rinsed with clean water several times until the smell of acetic acid and dye residue were removed. The resultant rinsed water was labeled as Effluent-2 solution; the MB dye concentration was found to be 2.9 mg L^−1^. Finally, the fiber was dried and checked for even dyeing.

## 3. Results and Discussion

### 3.1. Structural, Colloidal, Surface, and Magnetic Analysis

**Figure 1:**
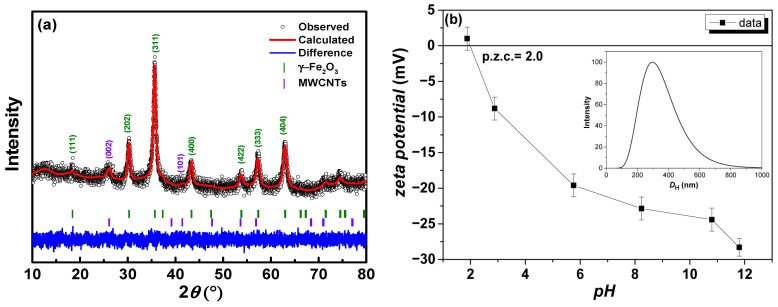
(**a**) Rietveld refined X-ray diffractogram. The black circles are experimental data, the red line is the calculated theoretical profile, the blue line is the residual, and each green Bragg peak represents the Miller indices on the top (olive for γ-Fe_2_O_3_ and violet for carbon). (**b**) pH dependence of the zeta potential for MWNT-NPE. The inset figure shows the hydrodynamic particle size distribution.

Figure 1a shows the Rietveld refined X-ray diffractogram of the MWNT-NPE sample. The crystallographic parameters are given in Table 1. First, it is worth noting that the use of MWCNTs and 5% *w*/*v Eucalyptus globulus* extract did not severely affect the crystallographic formation of 22 nm γ-Fe_2_O_3_ nanocrystallites.

When analyzing the titration curve of the MWNT-NPE sample, the point of zero charge (p.z.c.) was found to be ca. 2.0, indicating a predominance of negatively charged OH^−^ groups above this value. In contrast, a previous biosynthesized maghemite NP system depicts a p.z.c. of 3.9 [18]. On the other hand, as reported in the literature, the MWCNT surface is negatively charged when exposed to an acidic medium at 363 K [21]. In this case, there is not a reported p.z.c. value in comparison to the MWNT-NPE. In addition, it was found that a control 15 nm γ-Fe_2_O_3_ sample exhibited a p.z.c. of 7.0. Combining these results, it can be inferred that the presence of *Eucalyptus globulus* extract and MWCNTs significantly affects the disposable surface charge potential on MWNT-NPE and, therefore, increases its electrostatic affinity for the MB dye contaminant that usually shows a positive surface charge (cation-like structure).

**Figure 2:**
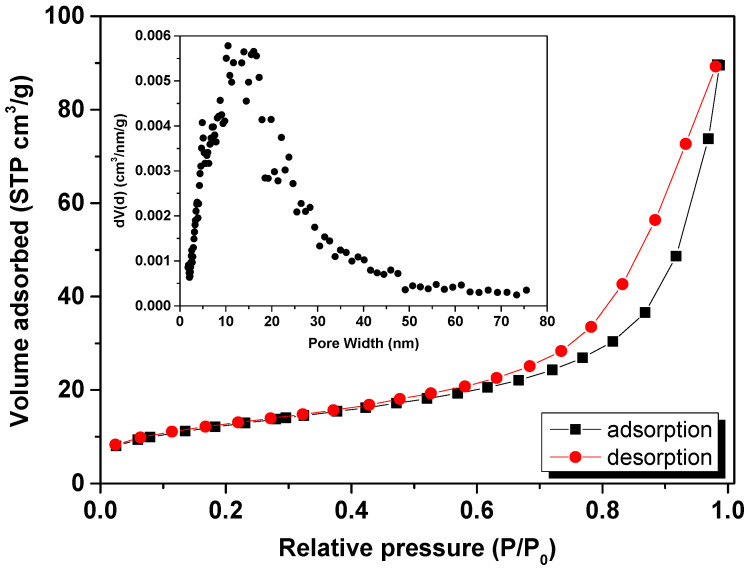
A 77 K N_2_ adsorption-desorption isotherm for the MWNT-NPE sample. The inset figure indicates the pore width distribution.

As can be seen in Figure 2, the 77 K N_2_-hysteresis adsorption–desorption isotherm represents an isotherm IV-type. This behavior is typically found in mesoporous systems [22]. The pore size distribution derived from the DFT approach exhibits uniform characteristics. The physisorption study indicates a BET surface area of 99 m^2^/g, a volume of 0.13 cm^3^/g, and a pore diameter of 12 nm.

**Figure 3:**
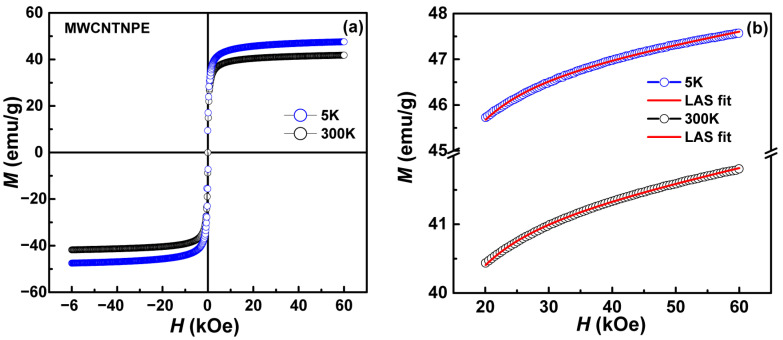
(**a**) *M*(*H*) curve for the MWNT-NPE sample recorded at 5 and 300 K. In (**b**), the red lines are results of fittings using the LAS equation for the data at 5 and 300 K.

Figure 3a depicts the *M*(*H*) loops recorded at 5 and 300 K for the MWNT-NPE sample. Their magnetic parameters were obtained after fitting the magnetization data to the Law of Approach to Saturation (LAS) equation [23]:

(3) $$M\left(H\right)=M_{S}\left(1-\frac{b}{H^{2}}\right)+\chi H$$

where *M*_s_ is the saturation magnetization, χ is the high-field susceptibility, and $K_{eff}$ is the effective anisotropy constant, calculated from $b=\frac{4}{15}\left(\frac{K_{eff}^{2}}{M_{S}^{2}}\right)$.

When looking at the fit magnetic parameters in Table 2, the 5 K *H*_c_ value agrees with those reported in other iron-based nanoxides [24,25]. Remarkably, the MWNT-NPE sample showed superparamagnetic behavior at 300 K (*H*_c_ = 0 kOe). In addition, it should be stressed that the *M*_S_ value decreases with temperature, as expected, but it is below the reported bulk iron oxides (60–90 emu/g) [26]. The reduction of magnetization can be intrinsically attributed to three main sources: (i) The presence of *Eucalyptus globulus* on the iron-oxide surface, (ii) the maghemite NP surface functionalization with MWCNTs, and (iii) the surface magnetic disorder (finite-size effect). The first two effects ((i) and (ii)) reduced the *M*_s_ value due to surface disorder introduced by non-magnetic materials [27], while the third one is basically due to the magnetic disorder on the iron-oxide NP surface. As is commonly observed in magnetic nanohybrids, the $K_{eff}$ values are one order above the bulk value, indicating an increase in the energy due to the functionalization process [18]. In addition, the *M*_r_/*M*_s_ ratio agrees with randomly oriented single-domain particle (i.e., *M*_r_/*M*_s_ ≤ 0.5) [18,24]

Despite not presenting systematic TEM images of our sample, the fingerprint structural, textural, and magnetic properties of γ-Fe_2_O_3_ nanophase, with a mean particle size < 20 nm, are contrasted with a previous system biosynthesized using *Citrus reticulata* peel residues [28].

### 3.2. Kinetic Adsorption Results

**Figure 4:**
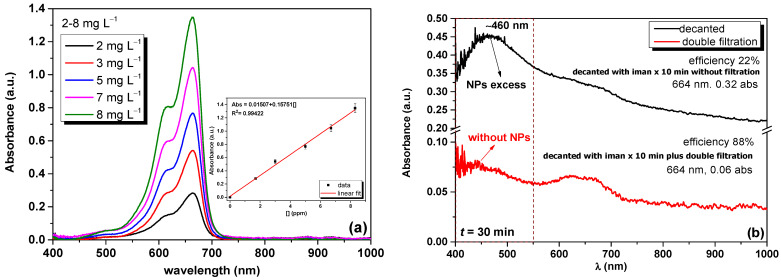

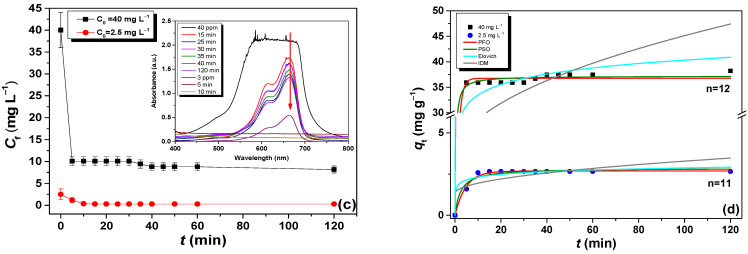
(**a**) MB calibration curve at MB concentration interval from 2 to 8 mg L^−1^, (**b**) preliminary absorbance band test for adsorption protocol improvement, and (**c**) time dependence of the MB concentration for two initial concentrations (the inset depicts the absorbance band intensity dependence with time and the arrow indicates the absorbance decrease). (**d**) Adsorbed amount as a function of time for tested initial concentrations. n-parameter indicates the number of experimental adsorption experiments that were carried out under the following conditions: 0.8 g L^−1^, pH = 6, and 300 K.

In the inset of Figure 4a, the calibration curve for MB solutions is depicted. An R^2^ value of 0.99422 was obtained from the linear fit. As mentioned in the experimental section, the absorbance band was correctly determined using a double-process filtration to eliminate the excess of NPs found at 460 nm; see Figure 4b and Figure S1 (decanted magnetic NPs after magnetic separation). In Figure S1, the removal efficiency achieved a value of 89% for 2.5 mg L^−1^ and 78% for 40 mg L^−1^ for an equilibrium time of 40 min. Certainly, this removal value can be improved with the adsorbent dose, as it will be shown ahead. More important, removing MB from influents at 10–200 mg L^−1^, as shown in real wastewater studies, is a suitable performance target for evaluating real-world applicability [29,30].

In Figure 4c, the MB concentration clearly decreases with time for both low and high initial concentrations. This is contrasted with the decrease in the absorbance bands (see the inset of Figure 4c). The presence of MB dimers [31] was evidenced by the saturated band at 40 mg L^−1^. The *q*_t_ was seen to increase with initial concentration, and the kinetic adsorption curves were tentatively fitted using four kinetic adsorption models, as shown in Figure 4d. The fitting parameters are given in Table 3 and Table 4, respectively.

From the kinetic adsorption analysis, at 2.5 mg L^−1^, the BIC parameter is close to 0 for the PSO, Elovich, and IDM (ΔBIC < 2.0). However, according to Lima et al. [32], this criterion must be accompanied by the R^2^ values discrimination. In that case, the suitable model corresponds to the PSO and Elovich models. In contrast, at 40 mg L^−1^, the data with the smaller BIC value is the PFO model with an acceptable R^2^ of 0.093. These results suggest that at low pH, the kinetic adsorption rate is dominated by a chemisorption process. On the other hand, at high concentration, the adsorption rate is basically governed by an entire physisorption process.

### 3.3. Adsorption Isotherm Analysis

**Figure 5:**
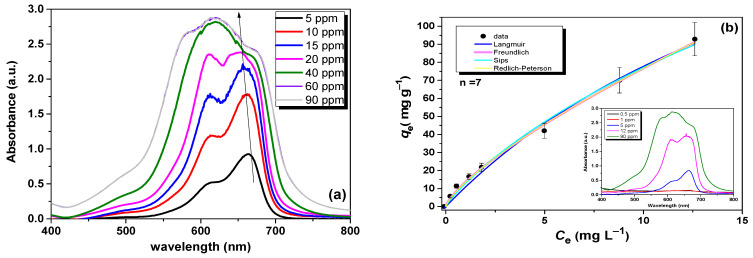
(**a**) Absorbance band intensity dependence with different ppm values of MB in the solution (The arrow indicates the formation of MB dimers). (**b**) Adsorption isotherm for MB using the MWNT-NPE sample for pH = 6, dose of 0.8 g L^−1^, and at 300 K. The data were fitted with different models with colors indicated in the inset.

In Figure 5a, the absorbance band intensity increased with different ppm values of MB in the solution, indicating an MB removal by the MWNT-NPE adsorbent. Figure 5b shows the adsorption isotherm at 300 K. An experimental *q*_e_ value of 92.9 mg g^−1^ was found for 40 min of exposure time. Four adsorption isotherm models were employed to analyze the experimental isotherm, and the obtained adsorption parameters are summarized in Table 5. Among all the models, the BIC parameter indicates that the Freundlich and Redlich-Peterson models are apparently the most adequate to describe the dynamic adsorption behavior (BIC close to 0). However, the difference between both BIC values, ΔBIC, is higher than 2.0 [32]. This result supports that the Freundlich isotherm model is the best to describe the adsorption isotherm data. It means that the adsorption process is governed by an MB heterogeneous adsorption on the MWNT-NPE sample.

### 3.4. Effect of pH

**Figure 6:**
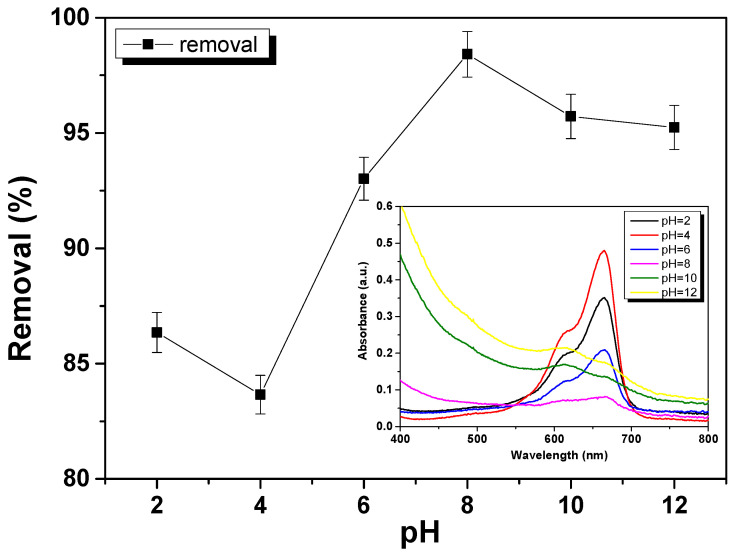
pH dependence of the removal efficiency. *C*_0_ = 20 mg L^−1^, adsorbent dose of 0.8 g L^−1^, equilibrium time of 40 min.

In Figure 6, the removal efficiency was studied as a function of the pH. At acidic pH (<6), the removal efficiency fluctuates between 84 and 87%, while at high alkaline pH (>7), the removal efficiency reaches 95%. The maximum value was found to be at pH = 8 with a 98% removal efficiency (considering the uncertainty region). These results agree with the titration curve shown in Figure 1b, where the isoelectric point was 2.0. The negative surface of the adsorbent, rich in OH^−^ groups, allows the electrostatic attraction for cationic MB dye with a slight decrease at high alkaline pH. This is due to the excess of hydroxide ions (OH^−^), which cause electrostatic repulsion between the negatively charged surfaces of the MB and MWNT-NPE nanohybrids after adhering to their surfaces. Under alkaline conditions, this repulsion lowers the equilibrium removal efficiency of MB (~95%). Therefore, the pH range of near-neutral to slightly alkaline is optimal for the MB adsorption process because it maximizes the electrostatic attraction between the negatively charged MWNT-NPE surface (~−20 mV) and the positively charged MB.

### 3.5. Adsorbent Dose Effect

**Figure 7:**
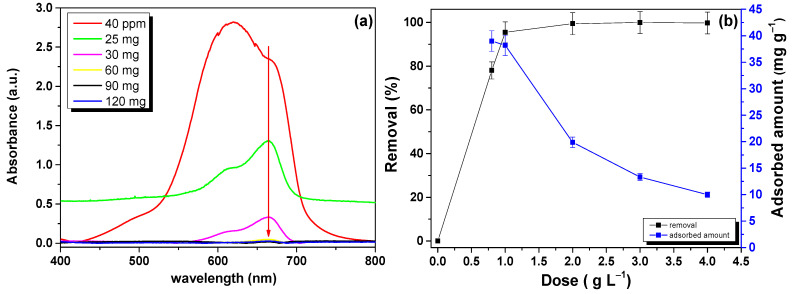
(**a**) Adsorbent dose dependence of the absorbance band intensity (the arrow indicates the absorbance decrease). For 60, 90, and 120 mg the curves are almost overlapped. (**b**) Adsorbent dose dependence of the removal efficiency and adsorbed amount. *C*_0_ = 40 mg L^−1^, equilibrium time of 40 min, and pH = 6.

In Figure 7a, the absorbance band intensity was seen to decrease with the increase of adsorbent dose. This was contrasted with the increase in the removal efficiency of MB at high adsorbent doses. From Figure 7b, a plateau was reached at 2 g L^−1^, reaching 99% removal efficiency. On the other hand, the adsorbed amount was observed to decrease, probably due to adsorbent surface saturation with the MB dye.

### 3.6. Comparison of Adsorption Parameters with Other Adsorbents

The application of magnetic nanoadsorbents for the MB removal has gained considerable attraction over the past five years, reflecting the synergy between high adsorption efficiency and the magnetic separability of these materials. Table 6 presents a comparative summary of seven distinct magnetic adsorbent systems investigated for MB removal, highlighting their physicochemical performance under various experimental conditions [33,34,35,36,37,38,39]. In terms of adsorption capacity, the highest reported value is 251.3 mg g^−1^ for Fe_3_O_4_/Activated Carbon [33], followed by WO_3_ nanoflakes that showed 38.52 mg g^−1^ [34] and BSA nanosorbent that displayed 38.52 mg g^−1^ [35]. These observed differences in adsorption capacity can be attributed to several factors, including surface area, porosity, and functional group availability. For example, WO_3_ nanostructures exhibited high surface area and active sites favorable for π–π interactions and the electrostatic adsorption of MB, while the proteinaceous surface of BSA nanosorbents may enhance dye binding through hydrogen bonding and hydrophobic interactions.

In addition, it should be mentioned that the removal efficiencies are generally high, with values exceeding 90% in the majority of cases. Notably, Mg@FA nanoadsorbent achieved up to 96% removal [36], underscoring the effectiveness of a composite system in enhancing MB adsorption through hydroxylated surface interactions. In contrast, BSA-based nanosorbents showed a lower removal efficiency (69%) [35], despite their moderate adsorption capacity, possibly due to their limited surface areas or aggregation effects during batch adsorption experiments.

On the other hand, considering that the adsorption process depends on several parameters, the role of some of them, like pH, equilibrium time, etc., on the results of MB removal will be discussed below. First, the solution pH plays a critical role in the adsorption process, for example, in the case of cationic dyes like MB. However, only a subset of studies reported this parameter, with optimal values generally falling between pH 6.5 and 11. This is consistent with the known behavior of MB, where higher pH promotes deprotonation of functional groups on the adsorbent surface, enhancing electrostatic attraction as observed in our system. Systems operating at neutral to alkaline pH, such as Mg@FA [36] and Fe_3_O_4_@MWCNT [37], are particularly attractive for practical applications due to their compatibility with typical wastewater conditions. Another important parameter to be analyzed is the equilibrium time that strongly affects the feasibility of large-scale adsorption processes. Among the studies that reported this parameter, one should point out the Fe_3_O_4_@MWCNT [37] and Mg@FA [36] that exhibited relatively long equilibrium times (120 min), but there is also other work, like BSA nanosorbents, that required only 60 min to reach equilibrium [35]. These variations are often governed by the porosity and diffusion path lengths in the adsorbent matrix. In our case, the equilibrium time of 40 min is very competitive with other nanoadsorbents, showing the potential of our material for applications in MB removals. Finally, the influence of the dose of adsorbent that ranged widely, from 0.02 g L^−1^, in highly efficient systems such as Fe_3_O_4_@MWCNT [37], to up to 10 g L^−1^, in less efficient systems like Mg@FA [36], will be discussed. This observation suggests a trade-off between intrinsic adsorption capacity and operational costs associated with the sorbent loading. The synergetic response of MWCNTs and iron-oxide NPs allows using intermediate doses with remarkable removal efficiency. In particular, initial MB concentrations, although underreported, are critical for the assessment of adsorption kinetics and capacity. Only the BSA nanosorbent study [35] clearly specified an initial MB concentration (100 mg L^−1^). This lack of standardization impairs cross-comparison and emphasizes the need for uniform reporting practices in adsorption studies. More importantly, the excess of initial MB concentration to only find and report high maximum adsorption capacities of a determined adsorbent implies excellent laboratory storage conditions for MB residues.

To conclude this section, it is important to address the limitations in data reporting. Several studies omit key parameters such as initial dye concentration, pH, or equilibrium time, which hinder complete quantitative comparison and meta-analytical assessment. Moreover, differences in synthesis methods, surface modification, and experimental protocols (e.g., agitation speed, temperature) must be acknowledged when interpreting the performance of these nanosystems.

### 3.7. Reuse Experiments

**Figure 8:**
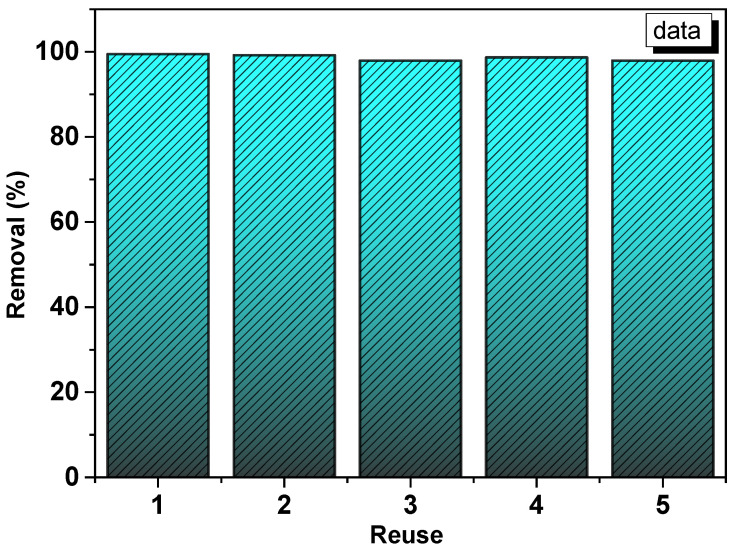
MB removal after five reuse experiments. *C*_0_ = 20 mg L^−1^, pH = 6, dose of 2 g L^−1^, 300 K, and equilibrium time of 40 min.

Reuse experiments were performed to corroborate the MB removal efficiency after five regeneration cycles, as shown in Figure 8. The removal efficiency was found to be above 98% under the tested repeated conditions. This result indicates that the MWNT-NPE adsorbent can be employed several times without losing its adsorption behavior.

### 3.8. FTIR Adsorption Mechanism

**Figure 9:**
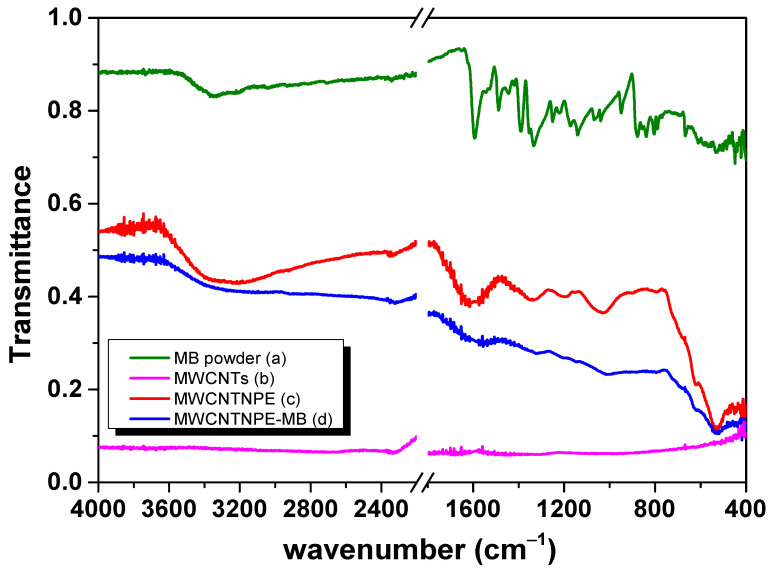
ATR-FTIR spectra for various samples (precursor materials and hybrid itself) studied in this work.

The FTIR analysis of methylene blue (MB) provides valuable insight into the vibrational characteristics of its functional groups and their interactions with the surrounding medium; see Figure 9a–d. MB, a heterocyclic aromatic compound with a phenothiazine core and dimethylamino substituents, exhibits well-defined infrared bands in the solid state, which can be directly associated with its structural features. In the solid state, the MB spectrum displays a broad band around 3440 cm^−1^, attributed to the stretching vibrations of O–H and/or N–H groups, which are typically associated with the intermolecular hydrogen bonding in hydrated crystalline forms of the dye [40,41]. The intense band around 1600 cm^−1^ is assigned to the stretching modes of aromatic C=C and imine C=N groups, which are conjugated within the phenothiazine skeleton. These features reflect the strong π-electron delocalization inherent to the dye’s chromophore. Additional characteristic peaks in the region of 1500–1400 cm^−1^ are attributed to aromatic C=C deformations, while the bands near 1250 cm^−1^ and 1220 cm^−1^ arise from C–N and N–N stretching, respectively [41]. The fingerprint region reveals several peaks, notably at 1176, 1146, and 1064 cm^−1^, which correspond to in-plane bending and mixed C–H/C–N/C–S vibrational modes. Finally, bands near 880 and 839 cm^−1^ are linked to out-of-plane aromatic C–H bending, confirming the planarity of the aromatic rings and their preserved symmetry [41]. The correlation between these vibrational modes and the molecular structure of MB makes FTIR spectroscopy a powerful tool for monitoring its state and interactions. Particularly, shifts in the C=N and C–N stretching regions serve as sensitive indicators of MB adsorption, protonation, or complexation with host materials. These spectral fingerprints are essential in adsorption studies and photocatalytic degradation research, where FTIR is frequently employed to confirm the presence or removal of MB on functionalized materials or nanoadsorbents [41].

In addition, the FTIR spectrum of MWCNTs was studied to have separate contributions that could maybe be found in the hybrid nanoadsorbent. Weak bands were observed mainly due to δ(OH^−^) at 1694 cm^−1^ and the one at ~1730–1770 cm^−1^ assigned to ν(C=O) [42]. Thus, considering that the FTIR spectrum of the MWCNT–COOH/*Eucalyptus globulus*/γ Fe_2_O_3_ nanohybrid displays well-defined bands at 3390–3185 cm^−1^, 1610 cm^−1^, 1340 cm^−1^, 1195 cm^−1^, 1023 cm^−1^, 623 cm^−1^, and 527 cm^−1^, it can be inferred for the synergistic integration of all components that form the hybrid nanoadsorbent. Specifically, it can be said that the broad band between 3390 and 3185 cm^−1^ is attributed to hydrogen-bonded O–H stretching, arising from the phenolic compounds in the *Eucalyptus globulus* extract and surface –COOH groups on the MWCNTs. Similar broad O–H features were reported in biosynthesized Fe_2_O_3_ NPs using *Eucalyptus globulus* leaf extract, where this band was linked to strong polyphenol-mediated capping of iron oxide NPs [43,44].

On the other hand, the intense peak at 1610 cm^−1^ corresponds to aromatic C=C stretching in the polyphenols and sp^2^ MWCNT backbone [45]. It also overlaps with asymmetric carboxylate stretching (ν_as_(COO^−^)), indicating coordination between Fe^3+^ and –COO^−^ moieties, consistent with spectral behavior in carboxyl-functionalized CNT–iron oxide composites [43]. Moreover, the medium-intensity band at 1340 cm^−1^ is assigned to symmetric carboxylate stretching (ν_s_(COO^−^)) or phenolic C–OH bending, an IR signature of chelated carboxylates in metal–carboxylate complexes [43], while the bands at 1195 cm^−1^ and 1023 cm^−1^ are characteristic of C–O–C and C–O [46] stretching from polyphenols, flavonoids, and glycosides present in the *Eucalyptus globulus* extract. Such features are common in plant-extract-synthesized nanomaterials [44,47]. Finally, the Fe–O vibrational modes at 623 cm^−1^ and 528–537 cm^−1^ are diagnostic of the γ Fe_2_O_3_ spinel lattice. Literature reports Fe–O stretching in magnetite/maghemite within the 550–625 cm^−1^ region and additional lattice modes at lower frequencies [43,44]

Overall, the FTIR profile confirms that the nanohybrid material is composed of a maghemite core stabilized by a bifunctional organic-inorganic matrix: The MWCNTs provide structural and conductive support, while the *Eucalyptus globulus* extract contributes surface-active groups capable of interacting with both the carbon nanotube surface and iron oxide particles. The presence of both coordinated carboxylate modes and Fe–O bands suggests strong chemisorptive interactions, likely through both coordination and hydrogen bonding. These interactions are critical for the stability, dispersibility, and potential functionality of the hybrid system, especially for applications involving pollutant adsorption, magnetic separability, and redox activity.

Now, considering the adsorption process of MB by the MWNT-NPE sample, it should be mentioned that the spectrum of the MWCNT–COOH/*Eucalyptus globulus*/γ Fe_2_O_3_ nanohybrid shows notable changes: (i) A broad band at 3299 cm^−1^, (ii) a shifted aromatic peak at 1553 cm^−1^, (iii) weak bands at 1320, 1206, and 1009 cm^−1^, (iv) altered Fe–O features appearing as a shoulder at 620 cm^−1^, and (v) a strong, intensified band at 535 cm^−1^. These observations clearly indicate MB adsorption via multiple chemical and physical interactions.

Specifically, it should be said that the broad absorption at 3299 cm^−1^ suggests the formation of hydrogen bonds between MBs –NH groups and surface –OH/–COOH functionalities of the nanohybrid. Similar spectral features have been reported in magnetic biochar systems used for MB adsorption, where strong O–H···N interactions were responsible for such band broadening [48]. On the other hand, the red-shifted aromatic stretch from ~1610 to 1553 cm^−1^ indicates π–π stacking interactions between MBs aromatic rings and the MWCNTs conjugated π-system, combined with electrostatic adsorption between MB^+^ and negatively charged carboxylate moieties. These shifts are characteristic of dye adsorption on magnetic Fe_3_O_4_/CNT composites, which display similar peak displacements [49]. Furthermore, the presence of weak bands at 1320, 1206, and 1009 cm^−1^ aligns with various C–N and possibly C–S vibrations of MB, confirming that the dye maintains its molecular integrity upon adsorption. These modes have also been observed in functionalized CNT–Fe_3_O_4_ systems after MB uptake [49]. These weak bands match the MB C–N vibrations and possibly C–O–C or C–OH modes of the extract. Their persistence suggests that MB has retained its molecular structure post-adsorption, and it is likely interacting with both MWCNTs and polyphenolic surface ligands. Finally, the modifications in the Fe–O region, particularly the shoulder at 620 cm^−1^ and intensified peak at 535 cm^−1^, reveal that MB adsorption perturbs the maghemite structure, likely through surface complexation with Fe^3+^ sites. Such shifts are consistent with literature reports of lattice vibration changes upon dye binding in magnetic nanocomposites [48].

**Figure 10:**
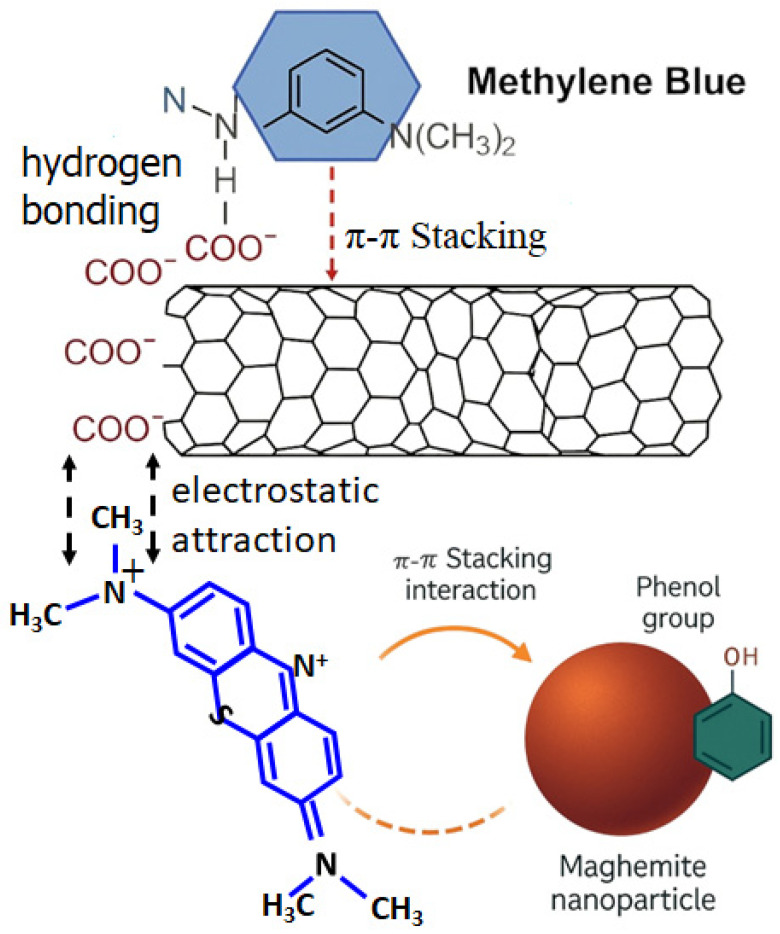
MB adsorption mechanism onto hybrid MWNT-NPE nanocomposite.

Overall, the FTIR data support a comprehensive adsorption mechanism involving (1) Hydrogen bonding between MB amino groups and composite hydroxyl/carboxyl sites, (2) π–π stacking interactions between MB and MWCNTs (or between phenol groups and MB), and (3) Electrostatic or coordination bonding between MB^+^ and Fe–O or carboxylate groups. In Figure 10, it is proposed that the adsorption mechanism occurred between the MB and MWNT-NPE samples, considering the results obtained from the FTIR analysis. Thus, it should be mentioned that these combined interactions contribute to the observed high adsorption efficiency, structural stability, and magnetic separability of the nanohybrid here produced. These characteristics make the material highly promising for dye remediation applications.

### 3.9. Application in a Real Effluent

**Figure 11:**
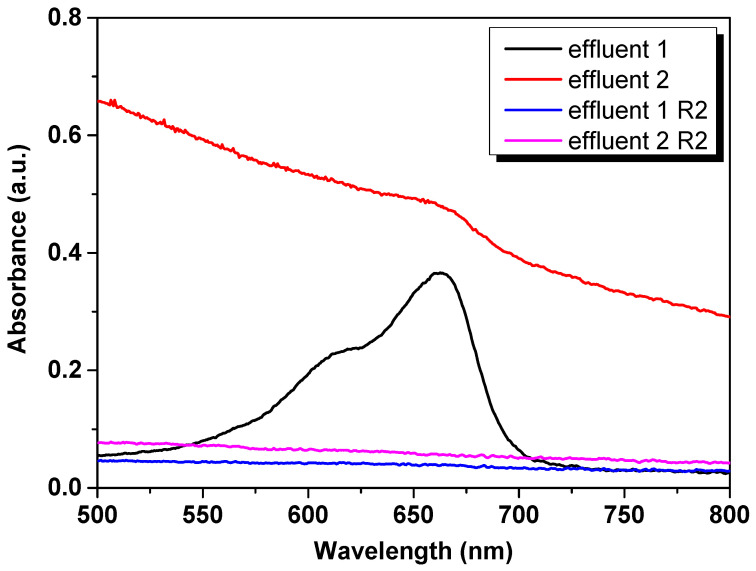
Absorbance bands obtained for the Effluent-1 and Effluent-2 (black and red lines). Absorbance lecturing after treating both effluents with MWNT-NPE nanohybrid. R^2^ indicates the second replicate.

Both Effluents-1 and -2 described in Section 2.4 with initial concentrations of 2.2 and 2.9 mg L^−1^ were exposed to an adsorbent dose of 0.8 g L^−1^, equilibrium time of 40 min, and initial pH of 6. From Figure 11, the UV-Vis absorbance measurements allowed calculating removal efficiencies of 92% and 97% for both effluents (obtained from two replicates). Hence, the MWNT-NPE nanohybrid can be used to treat effluents polluted with MB.

## 4. Conclusions

In this study, a magnetic nanohybrid, MWNT-NPE, was synthesized by decorating MWCNTs with γ-Fe_2_O_3_ NPs using *Eucalyptus globulus* extract. Structural and physicochemical characterizations confirmed a mesoporous feature of the nanohybrid with a surface area of 99 m^2^ g^−1^, an average pore diameter of 12 nm, and a mean nanocrystallite size of 22 nm. Magnetic measurements revealed superparamagnetic behavior at 300 K with an *M*_s_ value of 41 emu g^−1^, enabling efficient magnetic separation. The adsorbent achieved a maximum MB removal efficiency of 98% at pH 8 and a maximum adsorption capacity of 92.9 mg g^−1^ at 300 K. Kinetic experiments showed that equilibrium was reached within 40 min, with the adsorption process well described by the pseudo-second-order model at low MB concentrations (2.5 mg L^−1^) and by the pseudo-first-order model at higher concentrations (40 mg L^−1^). Freundlich isotherm modeling indicated a heterogeneous adsorption mechanism with high affinity (*k*_F_ = 14.1) and favorable conditions (1/n = 0.75). Mechanistically, FTIR spectra confirmed that adsorption was driven by a combination of π–π stacking between MB and the sp^2^-hybridized carbon lattice, electrostatic interactions between MB^+^ and negatively charged –COO^−^/–OH^−^ groups (consistent with a p.z.c. of 2.0), and hydrogen bonding facilitated by polyphenolic residues from *Eucalyptus globulus* extract. The role of the maghemite NPs extended beyond magnetic separability, providing Fe^3+^ coordination sites that contributed to dye binding via surface complexation. Importantly, this nanohybrid preserved > 98% adsorption efficiency over five regeneration cycles and achieved removal efficiencies of 92% and 97% for two synthetic textile effluents (MB ~2.2–2.9 mg L^−1^), underscoring its potential for real-world wastewater treatment. Together, these results validate MWNT-NPE as a synergistic, magnetically recoverable, and reusable adsorbent with a robust and multifaceted mechanism, offering a scalable and environmentally benign strategy for industrial dye remediation.

## Figures and Tables

**Table 1:** Rietveld structural and statistical parameters of refined crystallographic parameters for MWNT-NPE sample.

Phase	Atoms	Atomic Positions			Occ.	Lattice Parameters (nm)	Cagliotti Coefficients	Mean Size ± 1 (nm)	Statistical Parameters
		x	y	z					
$\gamma -Fe_{2}O_{3}$ $Fd\bar{3}m$ 86%	Fe-tet	0.1250	0.1250	0.1250	0.0386	a = b = c = 0.8348 α = β = γ = 90°	U = 0.2184 V = −0.0713 W = 0.1240	22	R_p_ = 60.5% R_wp_ = 28.6% R_exp_ = 27.4% $\chi^{2}$ = 1.09
	Fe-oct	0.5000	0.5000	0.5000	0.0683				
	O	0.2500	0.2500	0.2500	0.1730				
Carbon P63mc 14%	C1 C2	0.0000 0.3333	0.0000 0.6666	0.0000 0.0050	1.0000 1.0000	a = b = 0.2654 c = 0.6834 α = β = γ = 90°	U = 0.8011 V = −0.0891 W = 0.7974	Not calculated	

**Table 2:** Magnetic parameters obtained after fitting the *M*(*H*) curves with the LAS equation, considering high-field regions and temperatures at 5 and 300 K.

Sample	*T* (K)	*H*_C_ ±0.01 (kOe)	*M*_r_ ±0.1 (emu/g)	*M*_S_ ±0.1 (emu/g)	*K*_eff_ (×10^4^ J/m^3^)
MWNT-NPE	5	0.20	12.0	46.0	2.7
	300	-	-	41.0	2.1

**Table 3:** Kinetic adsorption parameters obtained from the nonlinear fittings to the raw adsorption data. *C*_0_ = 2.5 mg L^−1^.

	PFO Model	PSO Model
*q*_e_ (mg g^−1^)	2.68(3) × 10^0^	2.86(9) × 10^0^
*k*_1_ (min^−1^)	2.14(2) × 10^−1^	*k*_2_ = 1.4(4) × 10^−1^ (g mg^−1^ min^−1^)
R^2^	0.986	0.956
BIC	−19.47	−13.72
	**Elovich model**	**IDM**
α	$3(11)\times 10^{2}$ (mg g^−1^ min^−1^)	*k*_p_ = 2(13) × 10^−1^ (mg g^−1^ min^−1/2^)
β	4.1(1) × 10^0^ (g mg^−1^)	C = 1.3(4) × 10^0^ (mg g^−1^)
R^2^	0.910	0.425
BIC	−11.72	4.179

**Table 4:** Kinetic adsorption parameters obtained from the nonlinear fits to the raw adsorption data. *C*_0_ = 40 mg L^−1^.

	PFO Model	PSO Model
*q*_e_ (mg g^−1^)	3.68(3) × 10^1^	3.72(3) × 10^1^
*k*_1_ (min^−1^)	7.5(2) × 10^−1^	*k*_2_ = 9.7(4) × 10^−2^ (g mg^−1^ min^−1^)
R^2^	0.993	0.995
BIC	0.39	4.1
	**Elovich model**	**IDM**
α	2.3(0) × 10^4^ (mg g^−1^ min^−1^)	kp = 2.4(9) × 10^0^ (mg g^−1^ min^−1/2^)
β	3.35(5) × 10^−1^ (g mg^−1^)	2.1(5) × 10^1^ (mg g^−1^)
R^2^	0.969	0.356
BIC	37	62

**Table 5:** Adsorption isotherm parameters obtained from fittings of the data for the MWNT-NPE sample using nonlinear adsorption isotherm models for the MB.

	Langmuir Model		Freundlich Model
*q*_max_ (mg g^−1^)	2.5(7) × 10^2^	*k*_F_	1.4(1) × 10^1^
*k*_L_ (min^−1^)	4.6(2) × 10^−2^	1/n	7.5(3) × 10^−1^
R^2^	0.986	R^2^	0.996
BIC	71.34	BIC	61.68
	**Sips model**		**Redlich-Peterson**
*q*_s_	3.4(1) × 10^2^	*A*	9.8(0) × 10^1^
*K*_s_	4.0(0) × 10^−2^	*B*	6.3(5) × 10^0^
*m*	8.7(0) × 10^−1^	*β*	2.7(4) × 10^−1^
R^2^	0.992	R^2^	0.996
BIC	68.50	BIC	68.38

**Table 6:** Comparison of main adsorption parameters found in the literature for MB removal.

Adsorbent System	Adsorption Capacity (mg g^−1^)	Removal (%)	pH	Equilibrium Time (min)	Dose (g L^−1^)	References
Fe_3_O_4_/Activated Carbon	251.3	Not specified	6.5	60	0.1	[33]
WO_3_ Nanoflakes (green-synthesized)	78.14	Not specified	Not specified	24 h	Not specified	[34]
BSA Nanosorbent	38.52	69%	11	60	0.5	[35]
Mg@FA (Magnesium-modified fly ash)	14.34	95.61%	7–11	120	10	[36]
Fe_3_O_4_@MWCNT (sono-Fenton method)	Not specified	>90%	9	120	0.02	[37]
MNP-SDS	19.75	79%	5	Not specified	1	[38]
Modified Magnetic Corn Cobs (MCC)	13.23	>80% (alkaline pH)	7.69	10	4	[39]
MWNT-NPE	92.9	89%	6	40	0.8	This work

## Data Availability

The data related to this research can be requested at any time by sending an email to the corresponding author.

## Supplementary Materials

The following supporting information can be downloaded at: https://www.mdpi.com/article/10.3390/ma18133168/s1, Figure S1: Removal efficiency (%) vs. time (min) for both initial concentrations. 0.8 g L^−1^, pH = 6, 300 K; Section S1: Adsorption Kinetics and Isotherm Models; Section S2: Model Selection Criteria.
