# Supplementary material for: Biofunctional Magnetic Carbon Nanohybrid for Fast Removal of Methyl Blue from Synthetic Laboratory Effluent
Source: Materials (Basel). 2025 Jul 3;18(13):3168. doi: 10.3390/ma18133168 (PMC12251287; doi:10.3390/ma18133168)
Supplement: Supplementary file 1 [file materials-18-03168-s001.zip › materials-3730284-supplementary.pdf]

Supporting information for Biofunctional Magnetic Carbon Nanohybrid for fast removal of methyl blue from synthetic laboratory effluent

Juan A. Ramos-Guivar[1,*], Melissa-Alisson Mejía-Barraza[1], Renzo Rueda-Vellasmin[1], Edson C. Passamani[2]

[1]Grupo de Investigación de Nanotecnología Aplicada para Biorremediación Ambiental, Energía, Biomedicina y Agricultura (NANOTECH), Facultad de Ciencias Físicas, Universidad Nacional Mayor de San Marcos, Lima 15081, Peru

[2]Departamento de Física, Universidade Federal do Espírito Santo, Vitória 29075-910, Brazil

**Corresponding author: juan.ramos5@unmsm.edu.pe*

S1. Adsorption Kinetics and Isotherm Models

S1.1 Kinetic Models

- **Pseudo-First-Order (PFO) model:**

$$q_t = q_e \left(1 - e^{-k_1 t}\right) \tag{1}$$

where q_t and q_e are the adsorbed amounts at time t and equilibrium (mg g^{-1}), and k_1 is the rate constant (min^{-1}).

- **Pseudo-Second-Order (PSO) model:**

$$q_t = \frac{q_e^2 k_2 t}{1 + q_e k_2 t} \tag{2}$$

where k_2 is the PSO rate constant (g mg^{-1} min^{-1}).

- **Elovich model:**

$$q_t = \frac{1}{\beta} \ln(1 + \alpha\beta t) \tag{3}$$

where α is the initial adsorption rate (mg g^{-1} min^{-1}) and β is the desorption constant (g mg^{-1}).

- **Intraparticle Diffusion Model (IDM):**

$$q_t = k_p t^{1/2} + C \tag{4}$$

where k_p is the diffusion rate constant (mg g^{-1} min$^{-1/2}$), and C represents the boundary layer thickness (mg g^{-1}).

S1.2 Isotherm Models

- **Langmuir Isotherm:**

$$q_e = \frac{q_{\max} K_L C_e}{1 + K_L C_e} \tag{5}$$

where $q_{\max}$ is the maximum adsorption capacity (mg g^{-1}), K_L is the Langmuir constant (L mg^{-1}), and C_e is the equilibrium concentration (mg L^{-1}).

- **Freundlich Isotherm:**

$$q_e = K_F C_e^{1/n} \tag{6}$$

where K_F is the Freundlich constant ((mg g^{-1})(L mg^{-1})$^{1/n}$) and n is the heterogeneity index.

- **Sips Isotherm:**

$$q_e = \frac{q_s (K_s C_e)^m}{1 + (K_s C_e)^m} \tag{7}$$

where q_s is the maximum capacity, K_s is the equilibrium constant, and m is the heterogeneity factor.

- **Redlich–Peterson Isotherm:**

$$q_e = \frac{A C_e}{1 + B C_e^{\beta}} \tag{8}$$

where A, B, and β are fitting constants.

S2. Model Selection Criteria

S2.1 Residual Sum of Squares (RSS)

$$\text{RSS} = \sum_{i=1}^{n} (q_{e,\text{exp}} - q_{e,\text{calc}})^2 \tag{9}$$

where $q_{e,\text{exp}}$ and $q_{e,\text{calc}}$ are the experimental and model-predicted equilibrium adsorption values.

S2.2 Bayesian Information Criterion (BIC)

$$\text{BIC} = n \ln\left(\frac{\text{RSS}}{n}\right) + k \ln(n) \tag{10}$$

where n is the number of data points and k is the number of model parameters.

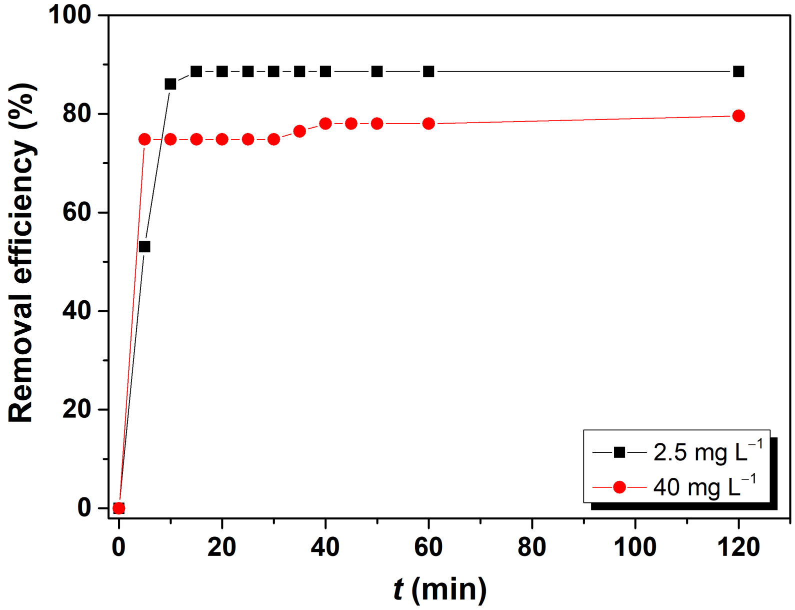

Figure 1: Removal efficiency (%) vs time (min) for both initial concentrations. 0.8 g L^{-1}, pH =6, 300 K.
